# Supplementary material for: Identification of photoperiod-induced specific miRNAs in the adrenal glands of Sunite sheep (Ovis aries)
Source: Front Vet Sci. 2022 Jul 22;9:888207. doi: 10.3389/fvets.2022.888207 (PMC9354845; doi:10.3389/fvets.2022.888207)
Supplement: Supplementary file 1 [file Data_Sheet_1.ZIP › Supplementary file/miRDeep2 file.zip!/miRDeep2 file/Know_Structure/SP42A1/oar-mir-412.pdf]

miRBase precursor : oar-mir-412
Total read count : 360
oar-miR-412-3p read count : 354
oar-miR-412-5p read count : 3
remaining reads : 3

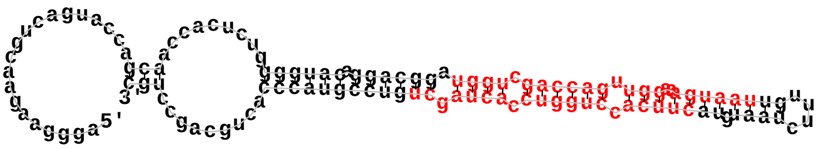

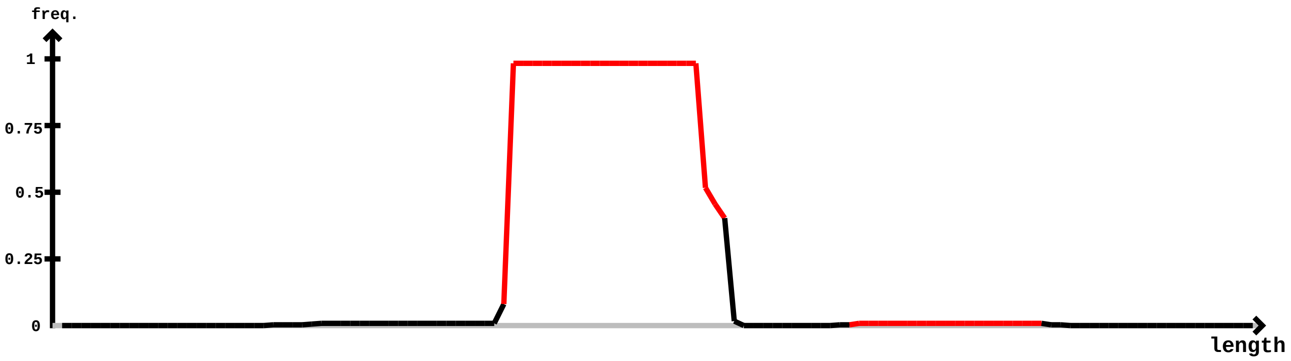

oar-miR-412-5p

oar-miR-412-3p

```
5'- agggaagaacgucaguaccagcaaccacucugggguacaggacggauggucgaccaguuggaaaguaauuguuucuaaauguacuucaccugguccacuagcuguccguacccacugcagccugc   -3'    exp
    ..........(.((((..(((........)))((((((.((((((.((((.((((((.(((..((((((((....)))).))))))).)))))).)))).)))))))))))))))))........    reads   mm
    .....................caaccacucugggguacaggacgga...............................................................................    1       0
    .........................cacucugggguacaggacgga...............................................................................    1       0
    ..........................acucugggguacaggacgga...............................................................................    1       0
    .............................................auggucgaccaguuggaaagu...........................................................    24      0
    .............................................auggucgaccaguuggaaagua..........................................................    1       0
    .............................................auggucgaccaguuggaaaguaa.........................................................    1       0
    ..............................................uggucgaccaguuggaaagu...........................................................    144     0
    ..............................................uggucgaccaguuggaaagua..........................................................    21      0
    ..............................................uggucgaccaguuggaaaguaa.........................................................    18      0
    ..............................................uggucgaccaguuggaaaguaau........................................................    139     0
    ..............................................uggucgaccaguuggaaaguaauu.......................................................    6       0
    .................................................................................uacuucaccugguccacuagcu......................    1       0
    ...................................................................................cuucaccugguccacuagcu......................    1       0
    ...................................................................................cuucaccugguccacuagcugu....................    1       0
```
